# Lifespan adversities affect neural correlates of behavioral inhibition in adults

**DOI:** 10.3389/fpsyt.2024.1298695

**Published:** 2024-01-16

**Authors:** Seda Sacu, Pascal-M. Aggensteiner, Maximilian Monninger, Anna Kaiser, Daniel Brandeis, Tobias Banaschewski, Nathalie E. Holz

**Affiliations:** ^1^Department of Child and Adolescent Psychiatry and Psychotherapy, Central Institute of Mental Health, Medical Faculty Mannheim, University of Heidelberg, Mannheim, Germany; ^2^Department of Child and Adolescent Psychiatry and Psychotherapy, University Hospital of Psychiatry Zurich, University of Zurich, Zurich, Switzerland; ^3^Neuroscience Center Zurich, University of Zurich and ETH Zurich, Zurich, Switzerland; ^4^Donders Center for Brain, Cognition and Behavior, Radboud University Nijmegen, Nijmegen, Netherlands; ^5^Department for Cognitive Neuroscience, Radboud University Medical Center Nijmegen, Nijmegen, Netherlands

**Keywords:** adverse experiences, early life stress, stop-signal task, fMRI, inhibitory control

## Abstract

**Introduction:**

Growing evidence suggests that adverse experiences have long-term effects on executive functioning and underlying neural circuits. Previous work has identified functional abnormalities during inhibitory control in frontal brain regions in individuals exposed to adversities. However, these findings were mostly limited to specific adversity types such as maltreatment and prenatal substance abuse.

**Methods:**

We used data from a longitudinal birth cohort study (*n* = 121, 70 females) to investigate the association between adversities and brain responses during inhibitory control. At the age of 33 years, all participants completed a stop-signal task during fMRI and an Adult Self-Report scale. We collected seven prenatal and postnatal adversity measures across development and performed a principal component analysis to capture common variations across those adversities, which resulted in a three-factor solution. Multiple regression analysis was performed to identify links between adversities and brain responses during inhibitory control using the identified adversity factors to show the common effect and single adversity measures to show the specific contribution of each adversity. To find neural correlates of current psychopathology during inhibitory control, we performed additional regression analyses using Adult Self-Report subscales.

**Results:**

The first adversity factor reflecting prenatal maternal smoking and postnatal psychosocial adversities was related to higher activation during inhibitory control in bilateral inferior frontal gyri, insula, anterior cingulate cortex, and middle temporal gyri. Similar results were found for the specific contribution of the adversities linked to the first adversity factor. In contrast, we did not identify any significant association between brain responses during inhibitory control and the second adversity factor reflecting prenatal maternal stress and obstetric risk or the third adversity factor reflecting lower maternal sensitivity. Higher current depressive symptoms were associated with higher activation in the bilateral insula and anterior cingulate cortex during inhibitory control.

**Conclusion:**

Our findings extended previous work and showed that early adverse experiences have a long-term effect on the neural circuitry of inhibitory control in adulthood. Furthermore, the overlap between neural correlates of adversity and depressive symptomatology suggests that adverse experiences might increase vulnerability via neural alterations, which needs to be investigated by future longitudinal research.

## 1 Introduction

Executive functions (EF) are essential cognitive skills for adaptation, social functioning, and goal-directed behavior (1). Deficits in EF have been documented in several psychiatric disorders, indicating that EF impairments may be a transdiagnostic correlate for psychopathology (2, 3). Previous findings showed that exposure to adverse childhood experiences is associated with both EF difficulties (4, 5) and poor mental health outcomes (6, 7), which lead to a developmental model suggesting that childhood adversities and other sources of stress may disrupt the neural systems supporting EF and thereby increase the risk of developing psychopathology (1). However, due to scarcity of longitudinal neuroimaging research, temporal dynamics of these relations have not been elucidated yet.

Inhibitory control is a core component of EF and a fundamental aspect of self-regulation, which requires suppressing a behavior or emotion when responding is no longer necessary or inappropriate (8). At the neural level, successful behavioral inhibition requires the involvement of several frontal regions, such as the inferior frontal gyrus, and the pre-supplementary motor area (9–13), whereas unsuccessful inhibition leads to enhanced activity in the dorsal anterior cingulate cortex (10, 13, 14), more likely reflecting error processing (15). Several studies found that individuals exposed to adverse childhood experiences showed altered frontal activation in these regions during inhibitory control tasks (16–22). However, the direction of the alterations changed based on the specific experimental paradigm used. Higher adversity was associated with higher activation in frontal regions in studies using the stop-signal task (16–19), whereas the reverse pattern was identified in the studies using the Go/No Go task (20–22). Although both Go/No Go and stop-signal tasks were commonly used in the context of response inhibition and require suppression of a dominant response, they involve distinct mechanisms, namely action restraint and action cancellation, respectively (23), which might explain conflicting directional associations found in previous literature.

Similar neural alterations, as found for adversities, were also identified in the context of psychopathology. Previous studies have reported abnormal prefrontal cortex activation during inhibitory control in several clinical conditions, including depression (24, 25), attention deficit and hyperactivity disorder (26), posttraumatic stress disorder (27), and eating disorder (28), indicating that altered frontal activation during inhibitory control can be a neural vulnerability correlate of psychopathology. However, the direction of the alteration was not consistent across the studies.

Although previous studies provided evidence for the relationship between childhood adversities and neural correlates of inhibitory control, there are several gaps in the literature that need to be addressed. First, the previous studies examined the effect of a single adversity measure on neural inhibitory network. However, it is plausible that different adversities could have common effects in addition to distinct associations with neural systems. Moreover, different adversities tend to occur together (29). Focusing on a single adversity measure may not only reflect the effect of specific adversity but also the effect of co-occurring adversities. Therefore, investigating shared effects as well as specific effects of diverse experiences will bring new insights. Second, most of the studies limited their findings to adolescent samples, except two previous studies conducted in adults using the Go/No Go paradigm (20, 22). Therefore, complimentary research is necessary to show if the long-term effect of adversities on neural inhibitory network is identifiable in other inhibitory control contexts. Third, most of the previous studies utilized liberal thresholds for reporting neuroimaging results (16, 17, 19, 21, 22), and some additionally investigated small sample sizes (16, 17, 19, 21).

The current study aimed to address these gaps by investigating the specific and cumulative effects of several lifespan adversities on neural responses during inhibitory control using the stop-signal task in a cohort of adults followed since birth. We collected several risk measures across development, which included prenatal factors such as maternal stress and maternal smoking, perinatal factors such as obstetric adversity, and postnatal factors such as low maternal care, family adversity, stressful life events, and self-reported childhood trauma. We hypothesized that adversities across development would be associated with increased functional activation in several frontal regions, including the inferior frontal gyrus (IFG), pre-supplementary motor area (pre-SMA), and dorsal anterior cingulate cortex (dACC) during inhibitory control. Furthermore, altered frontal activation would be associated with lower inhibition success and higher psychopathology symptoms in adulthood.

## 2 Methods

### 2.1 Participants

The current study was conducted within the framework of Mannheim Study of Children at Risk. The initial sample included 384 infants recruited from two obstetric and six children’s hospitals in the Rhine-Neckar region of Germany between 1986 and 1988. Participants were followed from their birth up to around the age of 33 years (age range: 31.7–34.5 years) across 11 assessment waves. At the last assessment wave (T11), 256 participants (67%) agreed to participate in the study and completed several psychological measurements. fMRI data for the stop-signal task was available for 170 participants. We used an extensive quality check procedure covering fMRI data quality (e.g., head motion, signal loss etc.), and task performance metrics based on a consensus guide for the stop-signal task (30) (see Supplementary material S1 for a detailed description of exclusion criteria). Four participants were excluded due to low fMRI data quality. An additional 45 participants were excluded due to poor task performance during go trials (correct go <80%), having inhibition success lower than 25% or greater than 75%, and having greater mean reaction time for unsuccessful stop trials than go trials. The final sample included 121 participants (Table 1). Of 121 participants, 16 participants fulfilled the criteria for a current psychopathology including major depressive disorder (*n* = 5), anxiety disorder (*n* = 7), alcohol and substance abuse (*n* = 3), and schizophrenia (*n* = 1).

**Table 1:** Sample characteristics.

	*N* = 121
Age, M (SD)	32.2 (0.3)
Sex, *N*, F/M	70/51
Head motion^a^, M (SD)	0.12 (0.05)
Maternal smoking, *N*, non−/moderate/heavy smoker	89/11/21
Maternal stress, M (SD), range	2.78 (1.9), 0–8
Obstetric adversity, *N*, no/moderate/high risk	41/72/8
Maternal stimulation^b^, M (SD), range	−0.27 (2.39), −7.18–5.96
CTQ total, Median (IQR), range	28 (5.5), 25–68
Family adversity, M (SD), range	3.39 (2.45), 0–10
Stressful life events^c^, M (SD), range	−0.56 (6.18), −11.23–22.27
ADHD, Median (IQR), range	4(6), 0–14
Antisocial personality, Median (IQR), range	2(4), 0–11
Anxiety, Median (IQR), range	3(3), 0–9
Avoidant personality, Median (IQR), range	2(4), 0–11
Depression, Median (IQR), range	3(5), 0–19
Somatic problems, Median (IQR), range	1(2), 0–11

a Frame-wise displacement. Measurement unit is millimeters.

b We used reversely-coded z-transformed scores. Higher scores indicated lower maternal stimulation.

c We used the sum score of z-transformed total scores across the 11 assessment waves.

### 2.2 Psychological measurements

#### 2.2.1 Adversity measurements

The Mannheim Study of Children Risk included several adversity measures across the development, which were previously associated with abnormal brain development and functioning (16, 20, 31–33). For the prenatal period, we included maternal stress (34) and maternal smoking (20), which were measured using a standardized interview during the 3-month assessment at T1. Obstetric adversity (35) included obstetric complications as a measure of perinatal risk. Postnatal measures included several psychosocial measures such as maternal stimulation (36) during infancy (3-month assessment), family adversity (37) from birth to up to 11 years (T5), stressful life events (38) from birth to up to around 33 years (T11), and self-reported childhood trauma at T9 (23 years) using the Childhood Trauma Questionnaire (CTQ) (39). Detailed descriptions for each adversity measure can be found in Table 2. Similar to our previous study (41), we applied a principal component analysis in IBM SPSS (version 27) using the above-mentioned adversity measures to reduce the dimensionality and account for correlative nature of the adversity measures (Supplementary Table S1). We identified three components with an eigenvalue >1, which in total explained 66.8% of the variance in the data (see details in Results).

**Table 2:** Adversity measures.

Measurement	Measurement time	Descriptions
Maternal smoking	T1 (3 months)	Maternal smoking measured daily cigarette consumption of mothers (1 = no, 2 = up to 5 per day, 3 = more than 5 per day) during pregnancy using a standardized interview.
Maternal stress	T1 (3 months)	Maternal stress was measured using a standardized interview. Mothers answered 11 questions covering negative experiences and reversely coded positive experiences during the second and third trimesters of pregnancy (e.g., “Did you have mood swings/ a depressed mode?”).
Obstetric adversity	T1 (3 months)	Obstetric adversity included obstetric complications (e.g., low birth weight, preterm birth, and medical complications). The score ranged between 0 and 4 (0 = no risk, 1–2 = moderate risk, 3–4 = high risk).
Maternal stimulation	T1 (3 months)	Maternal stimulation was based on video recordings of mother-infant interactions (10 min) in a play and nurse setting. Trained raters evaluated mothers’ attempts (vocal, facial or motor) to draw infants’ attention. The scores were z-transformed and recoded such that higher scores indicated lower maternal stimulation.
Family adversity	T1 – T5 (3 months – 11 years)	Family adversity measured the presence of 11 adverse family factors from birth to 11 years such as parental psychopathology, lower parental education, and marital discord.
Stressful life events	T1 – T11 (3 months – 33 years)	We measured stressful life events (e.g., presence of several life stressors in different domains such as partnership, education, work, health, and finance) across the development using an adapted version of the Munich Event List (38). The sum of Z-transformed scores calculated for each time point (T1–T11) was used for the analyses.
Childhood trauma	T9 (23 years)	Participants reported retrospectively the presence of traumatic childhood experiences using the German version of Childhood Trauma Questionnaire (40) covering five subscales (emotional abuse, emotional neglect, physical abuse, physical neglect, and sexual abuse). Total scores were used for the analyses.

#### 2.2.2 Psychopathology

We used the Adult Self-Report (42) to assess current symptoms of psychopathology. The Adult Self-Report includes 126 items rated on a 3-point Likert scale (0 = “not true”, 1 = “somewhat or sometimes true”, 2 = “very true or often true”) assessing mental health problems, adaptive functioning, and substance use. As measures of psychopathology, we used the total scores of six DSM-oriented subscales, including depression, anxiety, avoidant personality, somatic problems, attention deficit and hyperactivity disorder (ADHD) and antisocial personality scales.

### 2.3 Experimental paradigm

We used the stop-signal task (10) to assess inhibitory control during fMRI (Supplementary Figure S1). The task contained 160 trials (6.37 min), which consisted of two types of trials (go trials and stop trials). Each trial began with a fixation cross, which was followed by an arrow pointing to the left or right (go-signal). In the majority of trials (75%), participants were required to respond as quickly and accurately as possible by pressing the left or right button according to the previously shown arrow. Infrequently (25%), an arrow pointing upward (stop-signal) followed the go signal. During the stop trials, participants were asked to inhibit their response, which resulted in either successful or unsuccessful inhibition. The delay between go-signal and stop-signal started at 250 ms and increased by 50 ms if participants successfully inhibited their response (max 900 ms) or decreased by 50 ms if they failed (min 50 ms). This procedure enabled an approximately equal number of successful and unsuccessful stop trials. The inhibition success (successful stop trials / all stop trials) was on average 58.1% (SD = 8.9) in the current sample.

### 2.4 Data acquisition and preprocessing

The functional and structural images were acquired on a Siemens Magnetom Prisma Fit (Siemens, Erlangen, Germany) 3 T MRI scanner with a standard 32-channel head coil. During the stop-signal task, 186 volumes were obtained using a gradient echo-planar sequence sensitive to blood oxygen level-dependent (BOLD) contrast (36 slices, TE = 35 ms, TR = 2,100 ms, voxel size = 3 × 3 × 3 mm). More information on the scanning parameters can be found in the Supplementary material S4 (Supplementary Tables S2, S3).

Functional data was preprocessed using SPM 12.^1^ The first six volumes were discarded to allow for equilibration of the magnetic field. The preprocessing steps included slice timing correction of volumes to the middle slice, realignment to the first volume using a rigid body linear transformation, structural and functional image co-registration, segmentation, normalization to the Montreal Neurological Institute template, and smoothing using a kernel with a full-width half-maximum of 8 mm.

### 2.5 Generalized linear modeling

#### 2.5.1 First-level generalized linear modeling

Experimental conditions (correct go trials, successful stop trials, and unsuccessful stop trials) were convolved with a canonical hemodynamic response function using SPM 12. To quantify head motion, we calculated framewise displacement based on six motion parameters (43). If a participant had scans with framewise displacement greater than 0.5, we then censored those scans by creating a dummy-coded regressor (44). Six motion parameters, the regressor representing the censored scans, and time series from white matter and cerebrospinal fluid were entered into the first-level analysis as nuisance covariates to correct for motion and physiological noise. Having performed the first-level analysis, we created two widely used t-contrasts (10, 18, 45) in the literature: Successful stop trials > correct go trials and successful stop trials > unsuccessful stop trials.

#### 2.5.2 Second-level generalized linear modeling

All second-level analyses were conducted using SPM 12. We first performed a one-sample *t*-test to identify brain regions showing the main task effect. Results were thresholded at *p* < 0.05 (whole-brain family-wise error (FWE) corrected, cluster size >10).

We then conducted a series of multiple regression analyses to examine the association between three adversity factors and brain responses during inhibitory control on a whole-brain level. Sex and current psychopathology were included as covariates of no interest in all analyses. The same regression analysis was performed for each adversity measure separately.

In addition, we conducted regression analyses to explore brain-behavior relationship using task performance and psychopathology measures. We calculated several task performance metrics including the percentage of successful stop trials (i.e., number of correct stop trials / all stop trials) as a measure of inhibition success and stop-signal reaction time (SSRT) (46) as a measure of inhibition speed (47). Previous literature suggests that lower SSRT is related to higher inhibitory control (47). Unexpectedly, lower SSRT here was associated with higher commission errors during go trials (*r*_s_ = −0.27, *p* < 0.01), indicating that the higher the inhibition speed, the higher the commission error. This could be the case because healthy adults can develop a strategy (e.g., waiting longer) to increase inhibition success. Given the high correlation between the two metrics (*r* = 0.62, *p* < 0.001) and conflicting results regarding the SSRT, we opted to use inhibition success as a measure of task performance since inhibition success might be a more meaningful measure than inhibition speed in real-life settings. The results for the SSRT are presented in the Supplementary material S10.

To identify if there is an overlap between neural correlates of adversity and specific psychopathology, we first performed regression analyses using the above-mentioned six DSM-oriented Adult Self-Report subscales and then identified the regions showing both adversity and psychopathology effects by intersecting SPM whole-brain association maps.

All results were thresholded at a whole-brain level using *p* < 0.001 as a cluster-forming threshold, and the clusters with *p* < 0.05 corrected for FWE are reported in the results.

### 2.6 Statistical analyses

All statistical analyses were performed in IBM SPSS version 27. The analyses encompassed demographics for sample characteristics and correlation analyses to examine association the association between adversities and psychopathology. *P* was set to 0.05 (two-tailed). Due to non-normally distributed data for psychopathology measures (*n* = 6), we conducted a Spearman’s correlation test and applied Bonferroni correction to correct for multiple testing problem (*p* < 0.05 /6 = 0.008).

### 2.7 Post-hoc analyses

We identified adversity related alterations only during successful versus unsuccessful stop trials. However, this differential contrast did not reveal whether the activation difference was arisen due to more activation or less deactivation in one condition compared to other. Therefore, we further performed one sample *t*-tests using contrast images for successful stop trials (versus baseline) and unsuccessful stop trials (versus baseline). We then intersected the task and adversity effect maps obtained from second-level SPM analysis to identify regions showing shared effect.

Moreover, having identified the relationship between the first adversity factor and psychopathology measures, we conducted mediation analysis to see if neural responses mediates this relationship. For this purpose, we extracted mean activation from the clusters significantly related to adversity using the MarsBar toolbox.^2^ Mediation analysis was performed using the PROCESS toolbox (48) implemented in IBM SPSS version 27. In total, we tested 30 mediation models (five clusters x six psychopathology scales) using the model 4 from the PROCESS toolbox. To approximate the rigor of multiple comparisons correction, we set our confidence intervals to 99% and increased the number of bootstrap samples to 10,000. Each mediation model included mean activation from a cluster associated with adversity as a mediator (M) to explain the impact of the first adversity factor (X) on psychopathology symptoms (Y). In the current study, we used the sum scores of two psychosocial adversity measures that were assessed at multiple time points across development, namely family adversity (presence of 11 adverse family factors up to 11 years) and stressful life events (sum scores of the life events at each assessment wave up to 33 years). However, this approach does not allow to disentangle timing effects of adversities on neural systems. Given that the literature suggests that adverse experiences may exert more detrimental effects during specific developmental windows than the others (49), we performed regression analyses using the time-specific measures for family adversity (*n* = 5) and stressful life events (*n* = 11) (*p* < 0.001 at whole-brain, *p* < 0.05 FWE corrected at cluster level) in a further exploratory sensitivity analysis. All analyses were controlled for sex and current psychopathology. Due to the high number of tests and correlative nature, the findings should be considered preliminary.

## 3 Results

### 3.1 Behavioral results

The principal component analysis identified three adversity factors (Supplementary Table S4). The first adversity factor was strongly informed by stressful life events, family adversity, maternal smoking, and childhood trauma questionnaire. The second adversity factor was strongly related to obstetric adversity and maternal stress. The third adversity factor mostly reflected maternal stimulation. Similar to our previous work with a larger sample (50), we found that the first adversity factor was associated with higher scores in all psychopathology measures except for somatic problems (all *p* < 0.05, Bonferroni-corrected, Supplementary material S6). We did not identify any association between psychopathology measures and the second and third adversity factors, although the latter association was significant in the larger sample (50).

### 3.2 Task effect

**Figure 1:**
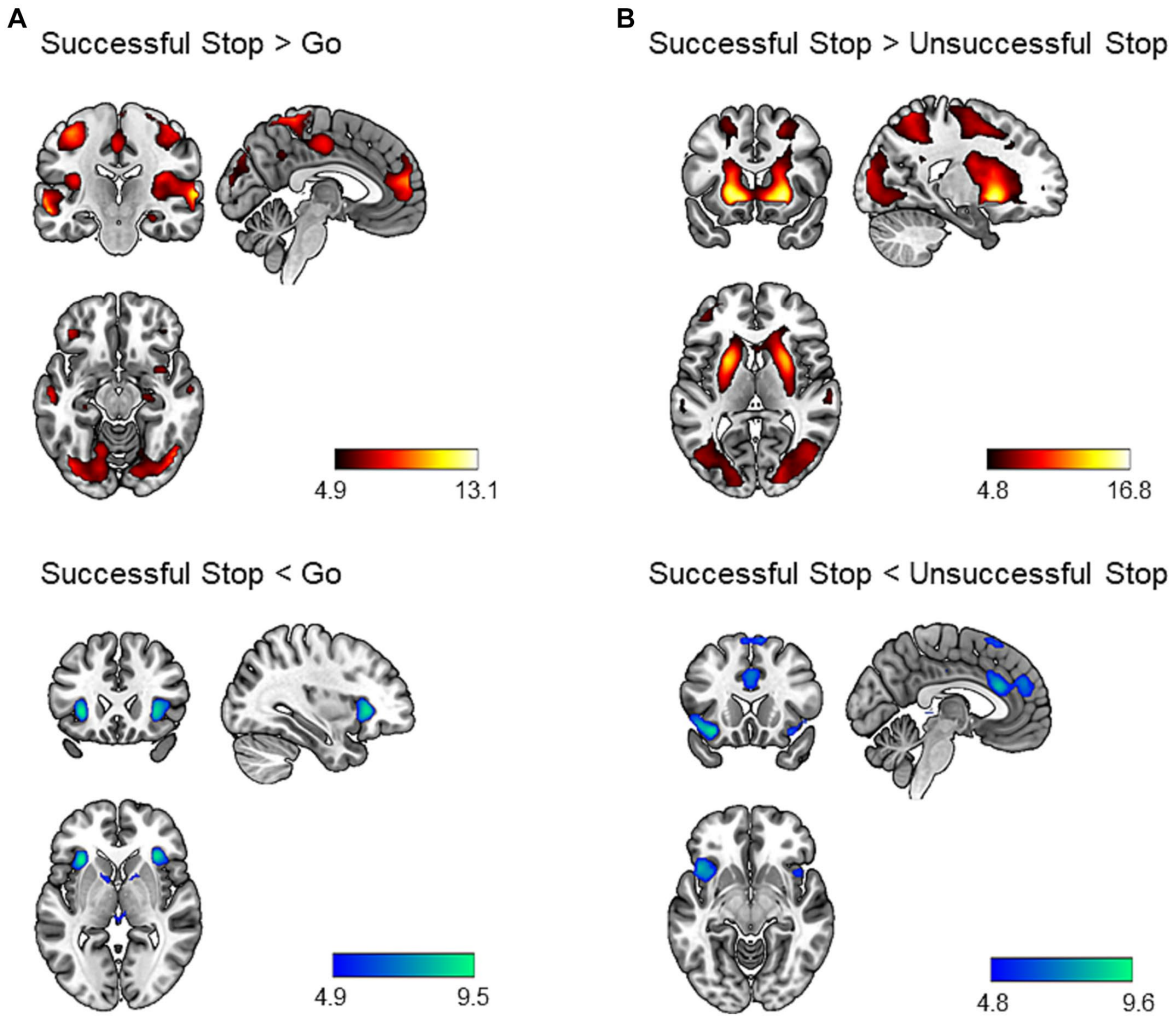
Brain regions showing task effect during the stop signal task (*p* < 0.05, whole-brain FWE corrected). Successful stop versus go trials **(A)** and successful stop versus unsuccessful stop trials **(B)** contrasts were chosen to identify neural correlates of inhibitory control. The hot colors represent increased activation, whereas the cold colors represent decreased activation for the contrast of interest. Results were mapped on the brain surface using MRIcroGL (https://www.nitrc.org/projects/mricrogl).

We performed a one-sample *t*-test to identify neural correlates of inhibitory control. The results are shown in Figure 1. During successful stop versus go trials, we found increased activation in several brain regions, including bilateral angular gyrus, middle temporal gyrus, cerebellum, precuneus, occipital regions, motor regions (precentral gyrus, postcentral gyrus, supplementary motor area), posterior insula, IFG, anterior cingulate cortex, orbitofrontal cortex, middle frontal gyrus, hippocampus, right amygdala, and left parahippocampal gyrus (*p* < 0.05, whole-brain FWE corrected; Supplementary Table S5). We additionally identified decreased activation in the bilateral anterior insula extending to the posterior IFG, bilateral putamen, and left midbrain (*p* < 0.05, whole-brain FWE corrected; Supplementary Table S5).

Similarly, during successful versus unsuccessful stop trials, we identified increased activation in a large cluster including bilateral occipital regions, striatum, frontal regions (middle, superior, and inferior frontal gyrus), motor regions (precentral gyrus, postcentral gyrus, SMA), middle temporal gyrus, superior temporal gyrus, precuneus, angular gyrus, amygdala, hippocampus, and left cerebellum (*p* < 0.05, whole-brain FWE corrected; Supplementary Table S5). In addition, we found decreased activation bilaterally in the anterior insula, orbitofrontal cortex, superior medial prefrontal cortex, dACC, and pre-SMA (*p* < 0.05, whole-brain FWE corrected; Supplementary Table S5).

### 3.3 Adversity effect

#### 3.3.1 Adversity factors

**Figure 2:**
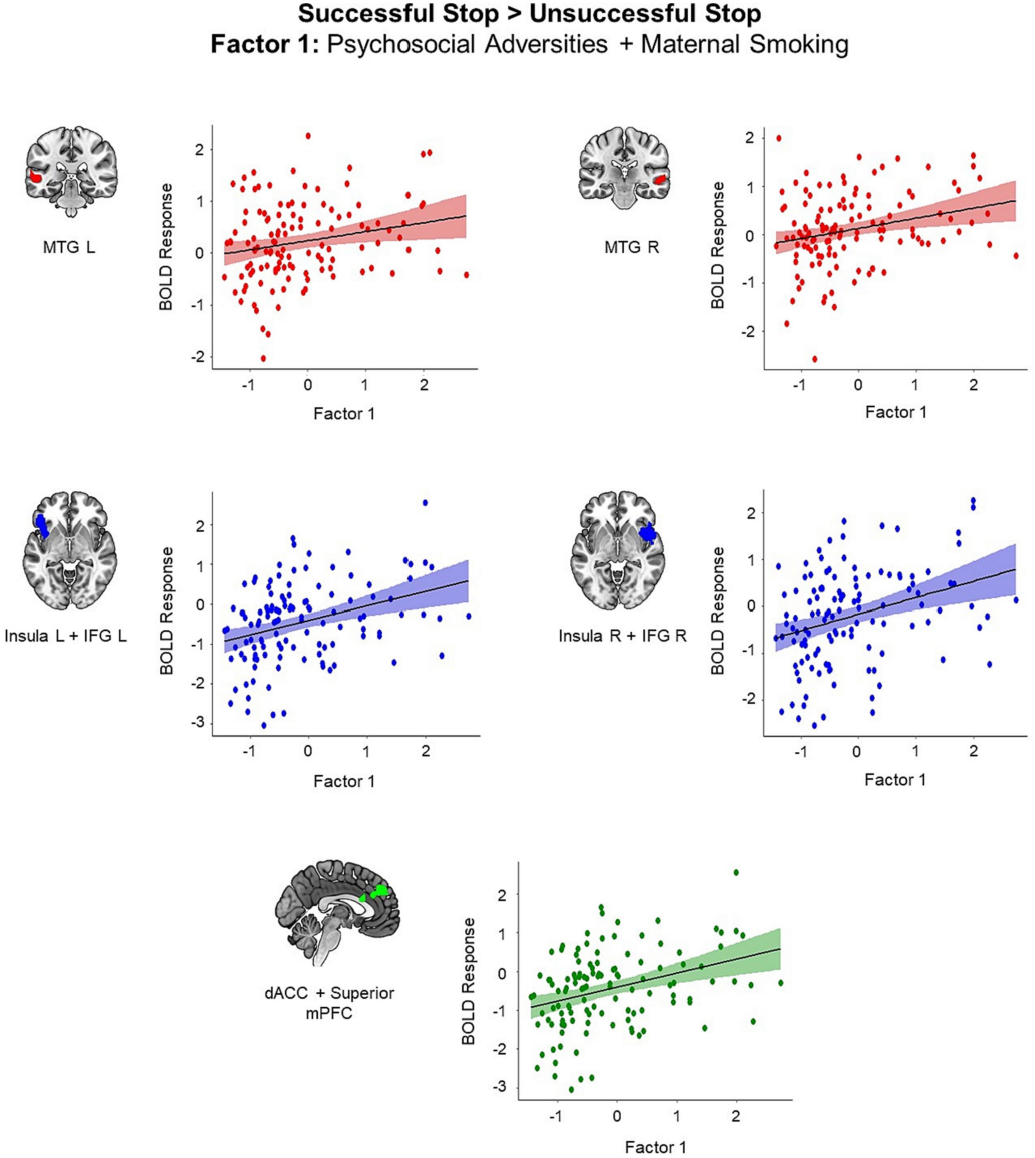
Brain regions showing positive associations with the first adversity factor during the successful versus unsuccessful stop trials (all *p* < 0.05, cluster-level FWE corrected). Scatter plots show the association between the scores in the first adversity factor and the mean BOLD response in the identified clusters for visualization purposes. dACC, dorsal anterior cingulate cortex; IFG, inferior frontal gyrus; mPFC, medial prefrontal cortex, MTG, middle temporal gyrus.

We identified five clusters showing positive correlation with the first adversity factor representing postnatal psychosocial adversities and prenatal maternal smoking (all *p* < 0.05, cluster-level FWE corrected; Figure 2). These clusters included left middle temporal gyrus (MTG) (*t* = 5.25, *k* = 95, *p* < 0.001), right MTG (*t* = 4.34, *k* = 64, *p* = 0.04), left insula extending to left IFG and left orbitofrontal cortex (*t* = 5.10, *k* = 259, *p* < 0.001), right insula extending to right IFG and right superior temporal gyrus (STG) (*t* = 4.78, *k* = 306, *p* < 0.001), and superior medial prefrontal cortex (mPFC) extending to dACC and middle cingulum (*t* = 5.19, *k* = 277, *p* < 0.001). Higher scores in the first adversity factor were associated higher activation in these regions during the successful versus unsuccessful stop trials.

We did not identify any cluster exhibiting correlation with the second and third adversity factors. Additionally, we did not identify any adversity-related alteration in brain activation for the successful stop versus go trials contrast.

#### 3.3.2 Specific adversity measures

**Figure 3:**
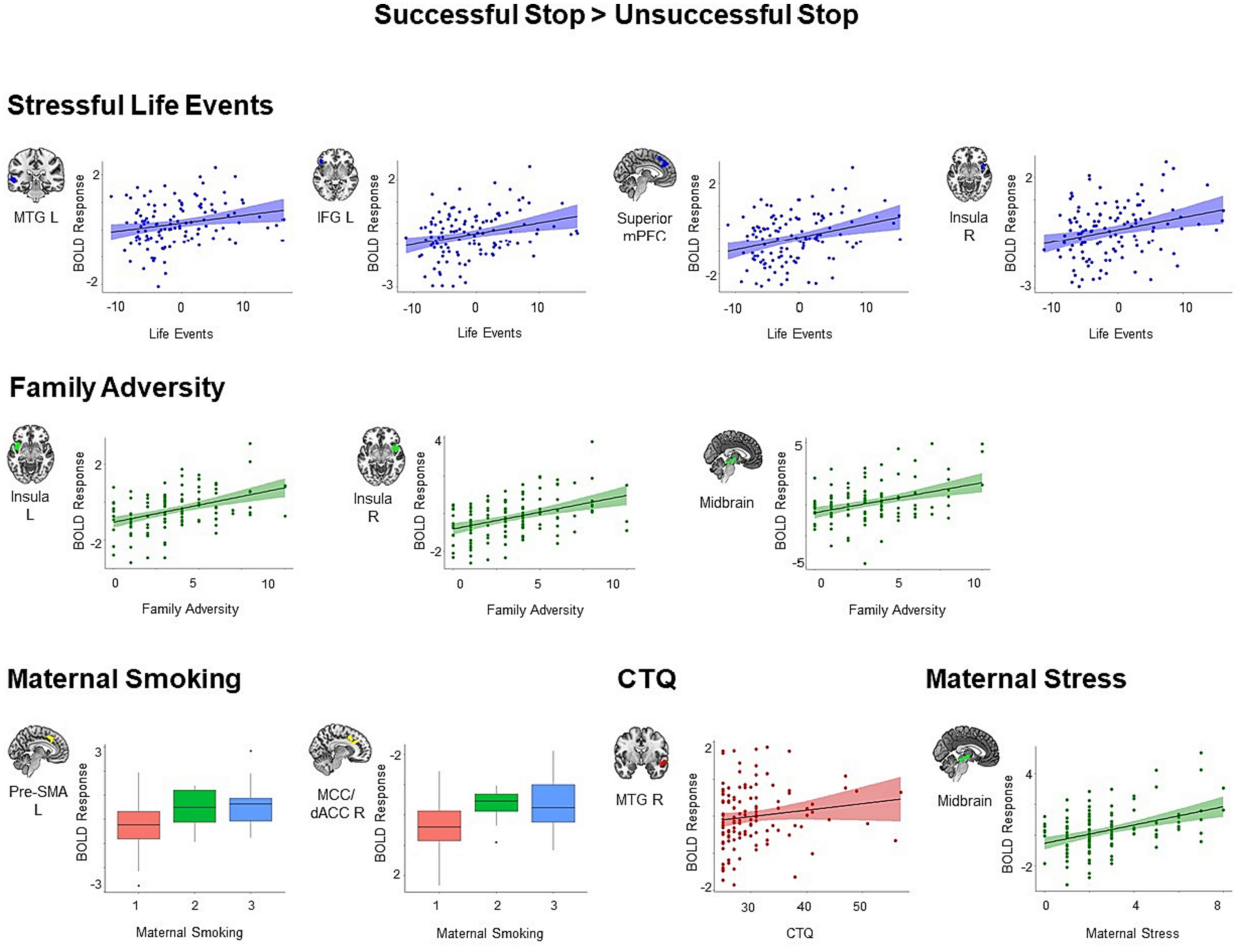
Positive associations between specific adversity measures and brain responses during inhibitory control (*p* < 0.05, cluster-level FWE corrected). Scatter plots show the association between the scores in the specific adversity measures and mean BOLD response in the identified clusters for visualization purposes. CTQ, Childhood Trauma Questionnaire; dACC, dorsal anterior cingulate cortex; IFG, inferior frontal gyrus; MCC, middle cingulate cortex; MTG, middle temporal gyrus; pre-SMA, pre-supplementary motor area.

We found similar results for the adversity measures constituting the first adversity factor, namely stressful life events, family adversity, prenatal maternal smoking and self-reported childhood trauma. All identified clusters showed increased activation during successful versus unsuccessful stop trials in individuals with higher adversity scores (all *p* < 0.05, cluster-level corrected, Figure 3). We identified four clusters that showed positive associations with stressful life events, including the left MTG (*t* = 5.13, *k* = 92, *p* = 0.01), right IFG extending to right orbitofrontal cortex, right insula, and right STG (*t* = 4.69, *k* = 113, *p* = 0.004), superior mPFC extending to left pre-SMA, middle cingulum, and left dACC (*t* = 4.40, *k* = 209, *p* < 0.001), and right insula (*t* = 4.22, *k* = 64, *p* = 0.04). Higher family adversity was linked to higher activation in the left insula extending to left IFG, left orbitofrontal cortex, and left STG (*t* = 5.20, *k* = 174, *p* < 0.001), right insula extending to right STG (*t* = 5.71, *k* = 179, *p* < 0.001), and midbrain (*t* = 4.20, *k* = 91, *p* = 0.01). Maternal smoking was related to higher activation in the left pre-SMA extending to left middle cingulum (*t* = 4.39, *k* = 88, *p* = 0.01), and right middle cingulum extending to right dACC (*t* = 4.34, *k* = 89, *p* = 0.01). Higher scores in the childhood trauma questionnaire were associated with higher activation in right MTG and STG (*t* = 4.45, *k* = 152, *p* < 0.001).

Moreover, maternal stress loaded to the second adversity factor was associated with higher activation in the midbrain (*t* = 4.14, *k* = 119, *p* = 0.003). We did not identify any significant cluster for lower maternal stimulation and obstetric adversity during successful versus unsuccessful stop trials.

Post-hoc analysis on the overlap between adversity and task effects (successful stop versus baseline, unsuccessful stop versus baseline, and successful stop versus unsuccessful stop) was reported in the Supplementary material S8.

Furthermore, our exploratory sensitivity analyses on the timing effect of adversities revealed that family adversity between the ages of 2 years and 11 years was related to higher activation in insula (all *p* < 0.05, FWE-corrected at cluster level; Supplementary Table S8 and Supplementary Figure S9). We found higher activation in MTG at T2, left insula\IFG at T5, and dACC at T7 and T9 during successful stop trials in response to stressful life events (all *p* < 0.05, FWE-corrected at cluster level; Supplementary Table S9 and Supplementary Figure S9).

### 3.4 Brain-behavior association

**Figure 4:**
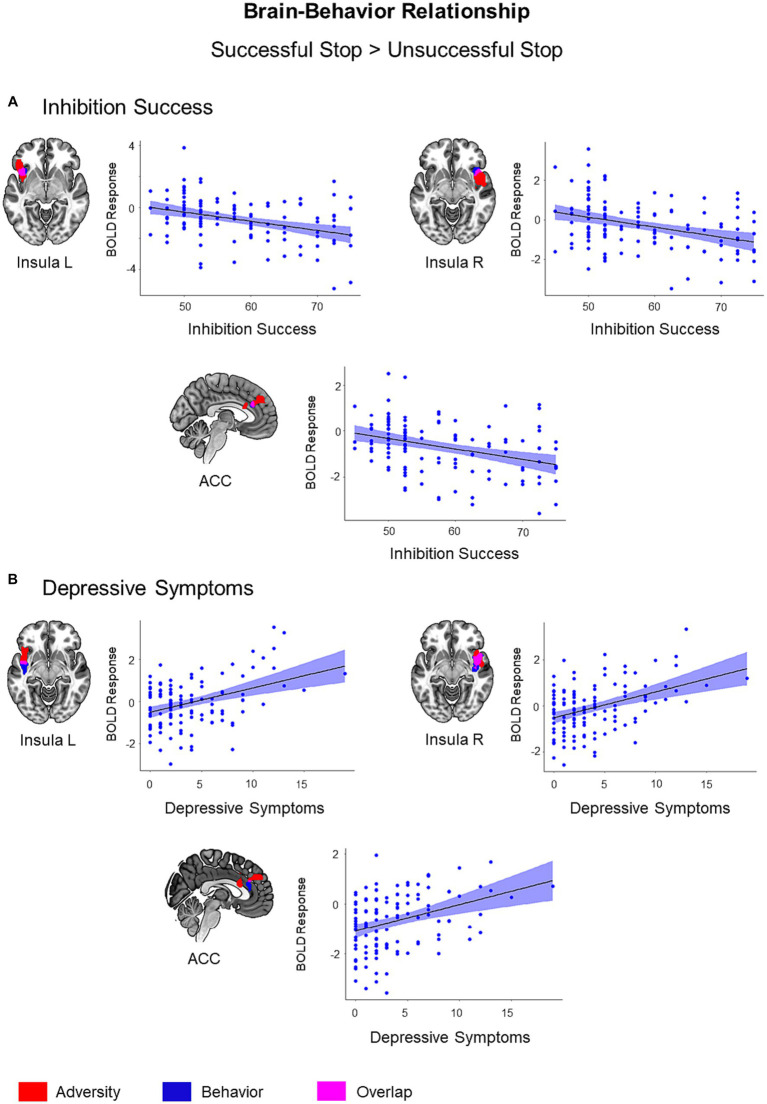
Brain-Behavior Relationship. Brain regions showing associations with inhibition success **(A)** and depressive symptoms **(B)** during successful versus unsuccessful stop trials were visualized in blue color on the brain surface (*p* < 0.05, cluster-level FWE corrected). Inhibition success showed a negative association with BOLD response, while depressive symptoms showed a positive association with BOLD response during successful versus unsuccessful stop trials. Adversity effect and overlap between adversity and behavior were visualized with red and pink color, respectively. Scatter plots show correlations between behavioral scores (inhibition success and depressive symptoms) and mean BOLD response extracted from the clusters associated with respective behavior (blue).

To explain the meaning of adversity related alterations, we conducted regression analyses with behavioral measures, a task performance measure (i.e., inhibition success) and psychopathology measures, using the successful versus unsuccessful stop trials contrast. Among the significant results, only the regions showing adversity effect were visualized in Figure 4 (*p* < 0.05, cluster-level FWE corrected).

#### 3.4.1 Inhibition success

Inhibitory control success was associated with lower activation in the left insula (*t* = 4.38, *k* = 69, *p* = 0.03), right insula (*t* = 4.38, *k* = 70, *p* = 0.03), and ACC (*t* = 4.13, *k* = 63, *p* = 0.04) during the successful versus unsuccessful stop trials. These regions also exhibited an overlap with the adversity effect (i.e., the first adversity factor) (Figure 4). Bilateral insula activation also showed an overlap with stressful life events and family adversity (Supplementary Figure S5). However, the overlap between adversity and lower inhibition success was more visible in the left insula. In addition, inhibitory control success was linked to higher activation in the left inferior occipital gyrus (*t* = 4.74, *k* = 425, *p* < 0.001), right inferior occipital gyrus (*t* = 4.43, *k* = 270, *p* < 0.001), right pre- and postcentral gyrus (*t* = 4.61, *k* = 444, *p* < 0.001), and precuneus/posterior cingulate cortex (*t* = 4.54, *k* = 272, *p* < 0.001).

#### 3.4.2 Psychopathology

Out of six psychopathology measures, only depressive symptoms were linked to higher activation in the right insula (*t* = 4.96, *k* = 260, *p* < 0.001), left insula (*t* = 4.55, *k* = 70, *p* = 0.03), and ACC (*t* = 3.87, *k* = 72, *p* = 0.03) during the successful versus unsuccessful stop trials. These regions also exhibited an overlap with the adversity effect (Figure 4). The effect of depressive symptoms overlapped with stressful life events and family adversity in bilateral insula (Supplementary Figure S6) and with inhibition success in the right insula and ACC (Supplementary Figure S7).

**Figure 5:**
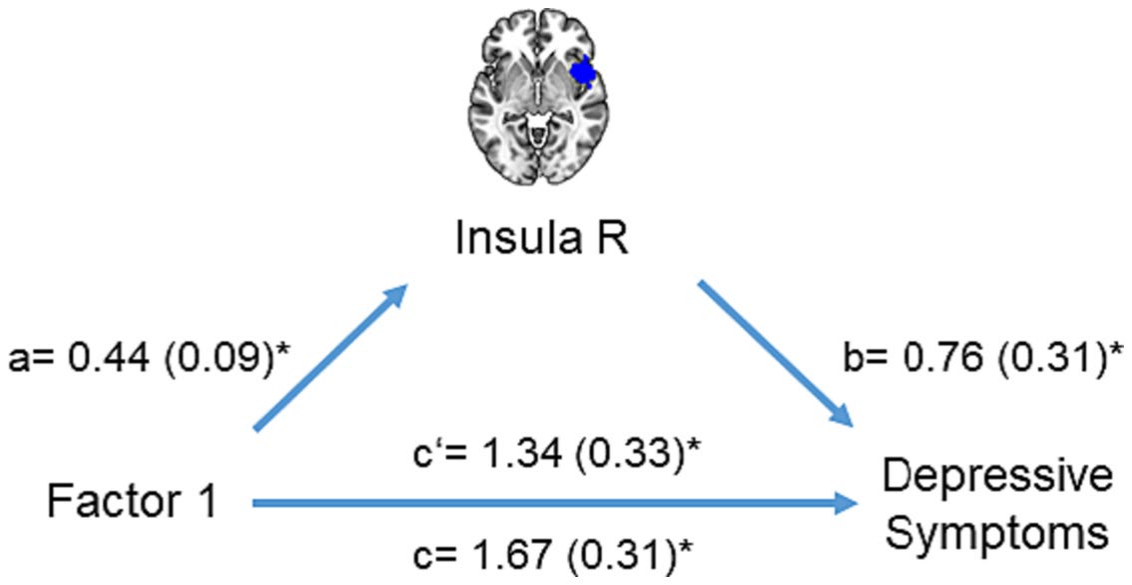
Mediation analysis. The mediation model included right insula activation as a mediator **(M)** to explain the impact of the first adversity factor **(X)** on depressive symptoms **(Y)**. Significant paths are shown with asterisk.

Furthermore, the mediation analysis revealed that right insula activation partially mediated the relationship between the first adversity factor and depressive symptoms (interaction effect (a×b) = 0.33, CI = [0.02 0.76], Figure 5). No other brain region mediated the relationship between the first adversity factor and depressive symptoms or other psychopathology symptoms.

## 4 Discussion

We here investigated the long-term effect of lifespan adversities on neural inhibitory network during the stop-signal task. Our results showed that lifespan adversities such as prenatal and postnatal psychosocial measures were associated with increased activation in several brain regions including IFG, dACC, insula, and MTG during successful versus unsuccessful stop trials in adults. Furthermore, increased activation in the insula and dACC was related to lower inhibition success and higher depressive symptoms. Taken together, our study contributes to the existing literature on adversity and inhibitory control by providing evidence for brain-behavior associations that were not explicitly demonstrated in previous studies. (16, 18, 19). Specifically, our findings regarding the stop-signal task and adversity-related neural alterations offer new insights into the neural mechanisms involved. This adds a novel dimension to our understanding of how adversity impacts brain function, particularly in the context of inhibitory control tasks.

The first adversity factor informed by postnatal psychosocial adversities and prenatal maternal smoking was associated with higher insula and dACC activation during successful compared to unsuccessful stop trials. In other words, individuals with higher adversity exhibited lower activation in these regions during the failed inhibition (i.e., unsuccessful versus successful stop trials). This effect was also partially overlapped with the task effect, where we found higher insula and dACC activation during failed inhibition across the participants (Supplementary material S8). Moreover, we found adversity-related neural alterations in both regions for stressful life events, only in insula for family adversity, and only in dACC for maternal smoking. Our exploratory analysis indicated an increased sensitivity of the insula for adversity during childhood and ACC during young adulthood. Taken together, these results indicate that insula and dACC activation were lower during failed inhibition in individuals with higher adversity with potentially different sensitive windows.

Insula together with dACC is a part of salience network and involves in error monitoring process (15, 51, 52). Previous studies utilizing directional connectivity methods reported a feedforward connectivity from the anterior insula to dACC following an error (51, 52), suggesting that the anterior insula might be involved in detecting saliency and signaling dACC that more attention is required to optimize a behavior after an error. Thus, reduced neural activation in the insula and dACC during failed inhibition might be related to reduced allocation of attention to errors in individuals with higher lifespan adversity, which in turn might lead to lower post-error behavioral adjustment. Indeed, we found that lower bilateral insula and dACC activation during unsuccessful versus successful trials were related to lower inhibitory control.

Furthermore, lower activation in the insula and dACC during failed inhibition was associated with higher depressive symptoms. Depression-related alterations also overlapped with the adversity effect. Several studies showed that depressed patients have difficulties in error monitoring (53), exhibit altered neural responses in dACC during error monitoring (24, 25), and abnormal resting-state salience network connectivity (54). Taken together, these results suggest that neural alterations in insula and dACC during error monitoring can be a potential vulnerability correlate for depressive symptoms and can be identified in non-clinical risk groups. Interestingly, although executive dysfunctions are assumed to be an important developmental pathway from early life stress to psychopathology in general (1) and previous studies have also identified error processing abnormalities in other clinical samples such as ADHD (26, 55), we only found an association with depressive symptoms. This might be related to our sample characteristics such as having lower variance in other psychopathology scales and a higher prevalence of depression in adulthood. Future research should also include clinical groups with adversity exposure to make inferences about the vulnerability aspect.

Higher scores in the first adversity factor as well as stressful life events and family adversity were related to higher IFG activation during successful versus unsuccessful stop trials. This result is compatible with a previous study showing higher IFG activation during inhibitory control in adolescents with early caregiver deprivation (16). Several studies found that IFG plays an important role in behavioral inhibition (12, 56). In line with the literature (9, 10, 14), we found that IFG was more active during successful stop trials compared to both go trials and unsuccessful stop trials across the participants. However, the adversity effect as well as inhibition success did not overlap with increased IFG activation (Supplementary material S8). Therefore, our results do not indicate that higher IFG activation in individuals with higher adversity is linked to higher inhibitory control. Moreover, enhanced IFG activation is not only found in inhibitory control tasks but also in several attentionally demanding tasks (57, 58). In addition, IFG activation is found to be modulated by task difficulty during the stop-signal task (59), suggesting higher IFG activation with more difficult stop trials. Indeed, a previous study showed that adolescents with prenatal alcohol exposure exhibited greater activation in several frontal regions with higher task difficulty (19). Taken together, higher IFG activation in individuals with higher lifespan adversity might be related to a compensatory recruitment due to higher attentional demand or task difficulty.

Additionally, we found that higher scores in the first adversity factor were related to higher activation in bilateral MTG during successful versus unsuccessful stop trials. This effect was also present for stressful life events and CTQ. MTG was more active during successful inhibition across the participants (Supplementary material S8), which is in line with other studies reporting increased activation in temporal regions during response inhibition (11, 60). However, none of the previous studies reported abnormal MTG activity related to adverse experiences during inhibitory control tasks, although altered MTG activation in relation to adversities was identified in other cognitive tasks such as sustained attention (61), working memory task (62, 63), and affective Stroop task (64). MTG is considered to be a part of the default-mode network and is associated with several cognitive functions including language processing, semantic memory and reasoning (65). However, due to a lack of behavioral associations and limited knowledge of its role in inhibitory control, it is difficult to explain why MTG activation is altered during inhibitory control in individuals with higher adversity.

In terms of specific adversity effects, most neural alterations were observed for stressful life events and family adversity. For maternal smoking, we identified an additional neural alteration in pre-SMA which was not identified in the common adversity factor. Higher maternal smoking was also related to higher dACC activation. With this finding, we replicated our previous work showing altered ACC activation in young adults exposed to prenatal maternal smoking during flanker/no-go task (20). Interestingly, higher total CTQ scores were only linked to higher MTG activation. We did not identify another region showing altered activation in relation to self-reported childhood trauma. However, these results must be interpreted with caution since the sample had low trauma exposure. Lastly, although the literature underscores the importance of parental behavior on cognitive development (66), we did not find any abnormal activation in individuals with lower maternal stimulation during infancy. However, our maternal sensitivity variable measures the socioemotional component of mother-infant interactions. Providing a cognitively rich environment (e.g., books and activities) can have different consequences on cognitive development than simply being emotionally available for the child. Therefore, the effect of cognitively stimulating home environment on executive functioning and brain responses should be further investigated by future studies.

To further address the reliability of our results, we investigated adversity-related neural alterations within the inhibitory control network using a longitudinal design that enables examination of long-term effects of adversities on adult brain functioning, a relatively large sample size, and a stringent methodological framework. The latter included comprehensive exclusion criteria for task performance and conservative thresholding, which collectively may enhance the generalizability and reliability of our findings. However, this study includes several limitations and needs to be interpreted with caution. First, although longitudinal studies offer valuable insights into how adverse experiences affect brain functions later in development, they do not provide enough evidence to make causal inferences. We here measured brain responses to inhibitory control only at the age of 33 years, whereas the adversity measures were collected across development. Thus, longitudinal neuroimaging studies are necessary and can offer a better understanding in terms of causality. Second, our exploratory analysis on the timing effect of psychosocial adversities should be interpreted with caution. Family adversity measures family characteristics that are tend to consistent across development, and it was not assessed beyond childhood. Except for stressful life events, our analysis did not include another adversity measure covering adolescence and adulthood periods. Third, our reliance on the Adult Self-Report for psychological assessments introduces potential biases inherent in self-report methods. These include recall bias, where participants may not accurately remember past events or feelings, recency bias, which might lead to overemphasis on recent experiences, and response bias, affecting the authenticity of the responses. We acknowledge these as critical limitations in interpreting our findings, given the retrospective nature of the data collected at each assessment wave. Fourth, we implemented principal component analysis to identify adversity factors that take into account the correlative nature of different adversity measures. Although it is helpful to model linear relations, non-linear relationships between variables could exist and be worth investigating. Therefore, future studies can implement machine learning approaches for clustering adversities to offer a better understanding of complex interactions between adversities.

In conclusion, our results indicated that higher psychosocial adversities and prenatal maternal smoking were linked to altered responses during successful versus unsuccessful stop trials in several brain regions that are important for successful response inhibition and error monitoring such as IFG, insula, and dACC. Lower insula and dACC activation during failed inhibition (i.e., unsuccessful versus successful stop trials) was further associated with lower inhibition success and higher depressive symptomology. Taken together, these results suggest that lifespan adversities are related to neural changes potentially heightening the risk of developing psychopathology. However, this aspect needs to be further examined by future studies using repeated prospective assessments of adversity and neural responses together.

## Data availability statement

The data analyzed in this study is subject to the following licenses/restrictions: Data available on request due to privacy/ethical restrictions. Requests to access these datasets should be directed to nathalie.holz@zi-mannheim.de.

## Ethics statement

The studies involving humans were approved by the local ethics committee of the medical faculty of the University of Heidelberg. The studies were conducted in accordance with the local legislation and institutional requirements. The participants provided their written informed consent to participate in this study.

## Author contributions

SS: Formal Analysis, Visualization, Writing – original draft, Writing – review & editing. PMA: Writing – review & editing. MM: Data curation, Writing – review & editing. AK: Writing – review & editing. DB: Conceptualization, Writing – review & editing. TB: Conceptualization, Funding acquisition, Resources, Supervision, Writing – review & editing. NEH: Conceptualization, Funding acquisition, Investigation, Supervision, Writing – review & editing.

## Footnotes

^1^ https://www.fil.ion.ucl.ac.uk/spm/software/spm12/

^2^ https://www.nitrc.org/projects/marsbar
