# Supplementary material for: Lifespan adversities affect neural correlates of behavioral inhibition in adults
Source: Front Psychiatry. 2024 Jan 16;15:1298695. doi: 10.3389/fpsyt.2024.1298695 (PMC10840329; doi:10.3389/fpsyt.2024.1298695)
Supplement: Supplementary file 1 [file Data_Sheet_1.docx]

**Supplementary Material**

**Lifespan adversities affect neural correlates of behavioral inhibition in adults**

Seda Sacu^1^, Pascal-M. Aggeinsteiner^1^, Maximilian Monninger^1^, Anna Kaiser^1^, Daniel Brandeis^1,2,3^, Tobias Banaschewski^1^, Nathalie E. Holz^1,4,5^

^1^ Department of Child and Adolescent Psychiatry and Psychotherapy, Central Institute of Mental Health, Medical Faculty Mannheim, University of Heidelberg, Mannheim, Germany

^2^ Department of Child and Adolescent Psychiatry and Psychotherapy, Psychiatric Hospital, University of Zurich, Zurich, Switzerland

^3^ Neuroscience Center Zurich, University of Zurich and ETH Zurich, Zurich, Switzerland

^4^ Donders Center for Brain, Cognition and Behaviour, Radboud University Nijmegen, Nijmegen, the Netherlands

^5^ Department for Cognitive Neuroscience, Radboud University Medical Center Nijmegen, Nijmegen, the Netherlands

Content

[S1. Data Quality Assurance 3](#_Toc153899260)

[S2. Correlations between Adversity Measures 4](#_Toc153899261)

[S3. Stop-Signal Task 5](#_Toc153899262)

[S4. MRI Scanning Protocol 6](#_Toc153899263)

[S5. Principal Component Analysis 7](#_Toc153899264)

[S6. Adversity Factors and Psychopathology 8](#_Toc153899265)

[S7. FMRI Task Effect 9](#_Toc153899266)

[S8. FMRI Task Effect: Post-hoc Analyses 10](#_Toc153899267)

[S9. Brain-Behavior Relationship 16](#_Toc153899268)

[S10. Neural Correlates of Stop Signal Reaction Time 17](#_Toc153899269)

[S11. Timing Effect of Adversities 18](#_Toc153899270)

# S1. Data Quality Assurance

fMRI data for the stop-signal task was available for 170 participants. Using an extensive quality check procedure, we excluded 49 participants. One participant was excluded due to high motion (> 3 mm in translation or > 3 degrees in rotation). Further three participants were excluded due to signal loss in the frontal cortex (n=1), incidental finding (n=1), and less number of volumes during the fMRI data acquisition (n=1). We also used a consensus guide for the stop-signal task to exclude the participants who did not meet behavioral performance requirements (1). One participant was excluded due to poor performance during the go trials (correct go < 80%). Twenty-six participants were excluded since their mean reaction time for unsuccessful stop trials was greater than the mean reaction time for go trials. Finally, we excluded 18 participants who had inhibitory control (i.e., successful stop trials / all stop trials) less than 25% or greater than 75%. The final sample size included 121 participants.

# S2. Correlations between Adversity Measures

**Table S1.** Correlations between adversity measures.

|  | Maternal Stress | Maternal Smoking | Maternal Stimulation | Obstetric Adversity | Family Adversity | Childhood Trauma Questionnaire | Stressful Life Events |
| --- | --- | --- | --- | --- | --- | --- | --- |
| Maternal Stress | - | 0.09 [b] | -0.04 [b] | **0.20*[b]** | **0.26** [b]** | 0.12 [b] | 0.15 [b] |
| Maternal Smoking |  | - | -0.01 [b] | -0.02 [b] | **0.32*** [b]** | 0.15 [b] | **0.39*** [b}** |
| Maternal Stimulation |  |  | - | -0.02 [b] | **-0.24**[a]** | -0.14 [b] | **-0.18*[a]** |
| Obstetric Adversity |  |  |  | - | -0.09 [b] | -0.02 [b] | -0.01 [b] |
| Family Adversity |  |  |  |  | - | **0.33*** [b]** | **0.56*** [a]** |
| Childhood Trauma Questionnaire |  |  |  |  |  | - | **0.41*** [b]** |
| Stressful Life Events |  |  |  |  |  |  | - |

* p < 0.05 ** p < 0.01 *** p < 0.001. a= Pearson’s correlation test, b= Spearman’s correlation test. Significant correlations are shown in bold font.

# S3. Stop-Signal Task

During the fMRI scan, participants completed a stop-signal task (2), which consisted of two types of trials: Go trials and stop trials. Each trial began with a 500 ms fixation cross. An arrow to the right or left (go-signal) was presented after the fixation cross. During most of the trials (75%), participants were required to respond to the arrow by pressing the right or left button based on the direction of the arrow in the present trial. Infrequently (25%), an upward arrow (stop signal) was presented following the go-signal. The delay between go signal and stop signal started at 250 ms and was adjusted based on participants’ performance. If participants correctly inhibited the response, the delay increased by 50 ms (max 900 ms), while it decreased by 50 ms if they failed to inhibit (min 50 ms). The task consisted of 160 trials, which approximately took 7 minutes.


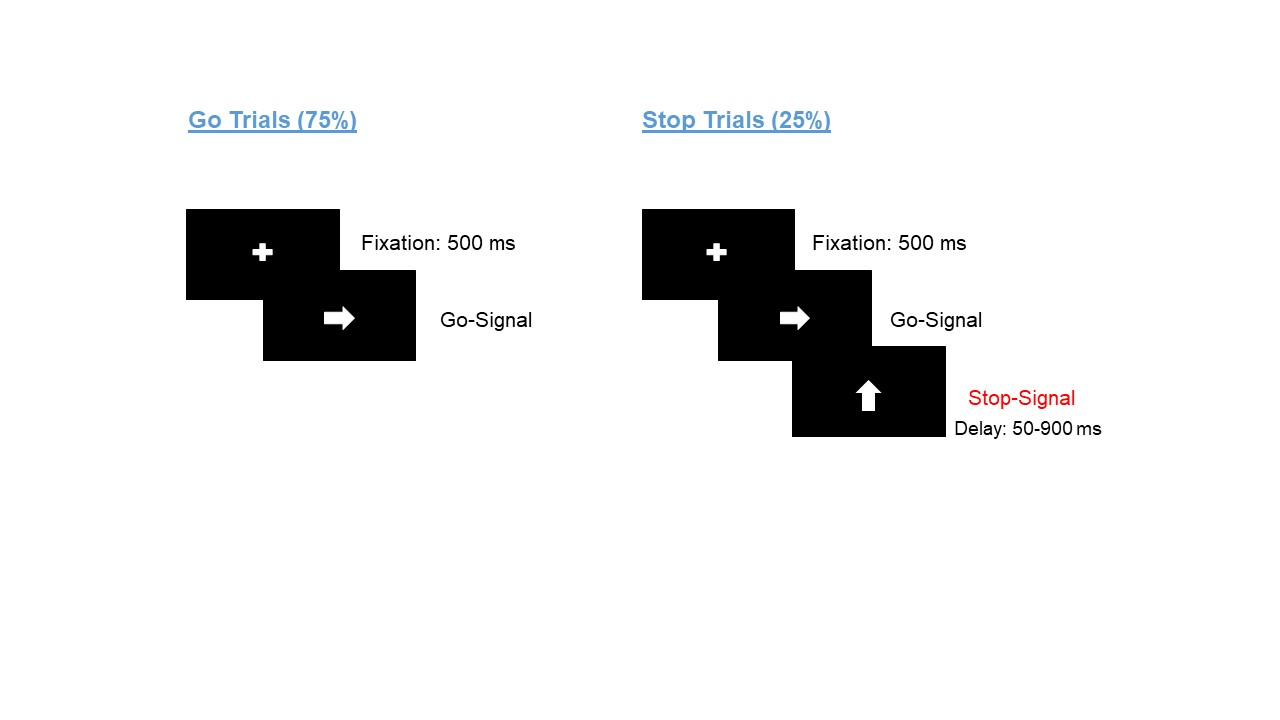


**Figure S1.** The Stop-Signal Task Design.

# S4. MRI Scanning Protocol

**Table S2.** Structural MRI scanning parameters.

| **Scanner** | **TR/TE/T1**  **(ms)** | **Flip angle** | **FOV** | **Matrix RL/AP/FH** | **Voxel size (mm)** | **Acceleration factor** |
| --- | --- | --- | --- | --- | --- | --- |
| Siemens Magnetom Prisma | 1800/2.6/900 | 8 | 230 | 350/263/350 | 0.9x0.9x0.9 | 2 |

**Table S3.** Functional MRI scanning parameters.

| **Scanner** | **TR/TE (ms)** | **FOV/ Flip angle** | **Slice number** | **Slice Order** | **Voxel Size (mm)** | **Total Acquisition Time**  **(min)** |
| --- | --- | --- | --- | --- | --- | --- |
| Siemens Magnetom Prisma | 2100/35 | 192/74 | 36 | Descending | 3x3x3 | 6.37 |

# S5. Principal Component Analysis

**Table S4.** The rotated component matrix for three-factor solution.

|  | Factor 1 | Factor 2 | Factor 3 |
| --- | --- | --- | --- |
| Maternal Stress | 0.26 | **0.76** | -0.02 |
| Maternal Smoking | **0.72** | 0.04 | -0.38 |
| Maternal Stimulation | 0.16 | 0.02 | **0.88** |
| Obstetric Adversity | -0.14 | **0.83** | 0.03 |
| Family Adversity | **0.80** | 0.10 | 0.13 |
| Childhood Trauma Questionnaire | **0.59** | 0.01 | 0.35 |
| Stressful Life Events | **0.83** | 0.02 | 0.19 |

# S6. Adversity Factors and Psychopathology

The first adversity factor informed by psychosocial adversities and prenatal maternal smoking was associated with higher scores in all psychopathology measures including ADHD (r_s_=0.32, p <0.001), anxiety (r_s_=0.42, p <0.001), antisocial personality (r_s_=0.29, p <0.001), avoidant personality (r_s_=0.31, p <0.001), depression (r_s_=0.49, p <0.001), and somatic problems (r_s_=0.20, p <0.05). All except the latter survived Bonferroni correction. The second adversity factor related to prenatal maternal stress and obstetric adversity was linked to only somatic problems (r_s_=-0.22, p <0.05), which did not survive Bonferroni correction. We did not identify any association between psychopathology and the third adversity factor informed by lower maternal stimulation.

# S7. FMRI Task Effect

**Table S5.** Brain regions showing task effect (p< 0.05, FWE-corrected).

| **Contrast** | **Direction** | **Peak Region** | **Cluster Size** | **MNI Coordinates** | | | **t** |
| --- | --- | --- | --- | --- | --- | --- | --- |
| StopS vs. Go | Positive | Angular Gyrus L | 8393 | -45 | -61 | 44 | 13.12 |
|  |  | MFG L | 1192 | -39 | 20 | 44 | 11.01 |
|  |  | OFC L | 97 | -42 | 35 | -13 | 8.35 |
|  |  | OFC R | 13 | 39 | 38 | -13 | 6.87 |
|  |  | MFG R | 17 | 42 | 50 | 11 | 6.42 |
|  |  | MFG L | 21 | -39 | 50 | 5 | 6.31 |
|  |  | PHG L | 19 | -30 | -25 | -16 | 6.15 |
|  |  | ACC R | 29 | 9 | 35 | -7 | 5.88 |
|  | Negative | Insula L | 145 | -33 | 21 | -1 | 9.52 |
|  |  | Insula R | 149 | 33 | 26 | -1 | 9.20 |
|  |  | Midbrain L | 74 | -3 | -31 | -1 | 7.23 |
|  |  | Putamen R | 37 | 18 | 8 | -4 | 7.04 |
|  |  | Putamen L | 45 | -18 | 8 | -4 | 6.69 |
|  |  | IFG R | 61 | 39 | 8 | 26 | 6.52 |
| StopS vs. StopU | Positive | Putamen L | 13004 | -24 | 8 | -1 | 16.79 |
|  |  | STG L | 132 | -57 | -7 | -4 | 7.33 |
|  |  | STG R | 132 | 63 | -22 | -4 | 7.32 |
|  |  | Cerebellum R | 110 | 42 | -67 | -34 | 7.29 |
|  |  | Lingual L | 12 | -18 | -43 | -7 | 5.38 |
|  | Negative | Insula L | 166 | -33 | 17 | -13 | 9.57 |
|  |  | ACC | 359 | 0 | 26 | 26 | 9.24 |
|  |  | SMA R | 70 | 9 | 20 | 65 | 7.02 |
|  |  | Insula R | 43 | 33 | 17 | -13 | 6.17 |
|  |  |  |  |  |  |  |  |

**Abbreviations:** ACC, anterior cingulate cortex; IFG, inferior frontal gyrus; MFG, middle frontal gyrus; OFC, orbitofrontal cortex; PHG, parahippocampal gyrus; SMA, supplementary motor area; STG, superior temporal gyrus; StopS, stop successful; StopU, stop unsuccessful.

# S8. FMRI Task Effect: Post-hoc Analyses

**Anatomical Specification of Brain Regions Showing Mixed Activation Pattern**

We reported both increased and decreased activation in insula and inferior frontal gyrus for the successful stop versus go trials contrast (See Figure S2A). Anterior insula (Figure S2A, left panel) showed decreased activation during the successful stop trials compared to the go trials. The same activation pattern can also be seen in the contralateral site. These clusters included mostly insula but also inferior frontal gyrus to a smaller extent, posteriorly located in the frontal cortex. In contrast, posterior insula (Figure S2A, middle panel) showed increased activation during the successful stop trials compared to the go trials. Increased activation in the inferior frontal gyrus cluster was anteriorly located and separated from the insula clusters (both anterior and posterior insula clusters; Figure S2A, right panel).

For the successful stop versus unsuccessful stop contrast, we similarly found decreased activation in anterior insula (Figure S2B, left panel). The supplementary motor area showed both increased and decreased activation. Increased activation was located posteriorly (Figure S2B, middle panel), whereas decreased activation was located anteriorly (Figure S2B, right panel).

**
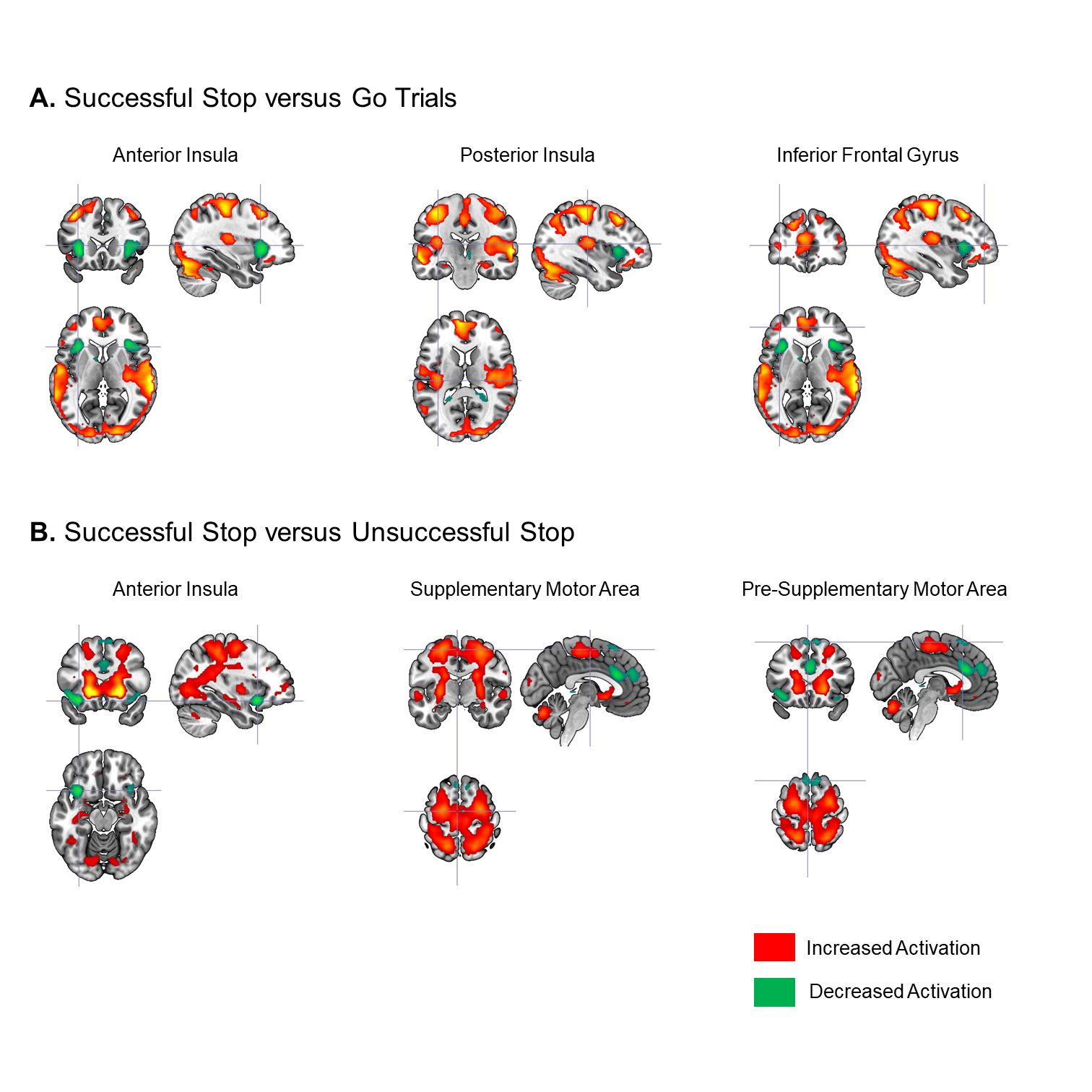
**

**Figure S2.** Anatomical specification of brain regions showing both increased and decreased task activation.

**Interpretation of Differential Task Effect**

Since we identified adversity related brain alteration during successful stop versus unsuccessful stop trials, we wanted to identify brain activation pattern during successful stop > baseline and unsuccessful stop > baseline contrasts to be able to interpret the results for the differential contrast. All results were corrected for family-wise error at whole brain level (p < 0.05).

During successful stop trials compared to baseline, we found enhanced activation in cerebellum, occipital cortex, precentral and postcentral gyri, supplementary motor area, inferior parietal cortex, several temporal regions (middle temporal gyrus, superior temporal gyrus), middle and superior frontal gyri, insula, and striatum. At uncorrected level, we also identified increased activation in the inferior frontal gyri, especially in the left hemisphere. We also found decreased activation in insula, lingual gyrus, fusiform, parahippocampal gyrus, cuneus, precuneus, brainstem, and right inferior frontal gyrus (Figure S3).

During unsuccessful stop trials compared to baseline, we found enhanced activation in dorsal anterior cingulate cortex, superior medial frontal cortex, supplementary motor area, middle temporal gyrus, insula, fusiform gyrus, and left inferior frontal gyrus. We also identified decreased activation in middle temporal gyrus, occipital cortex, striatum, insula, inferior frontal gyrus, precuneus, postcentral gyrus, and middle and superior frontal gyri.


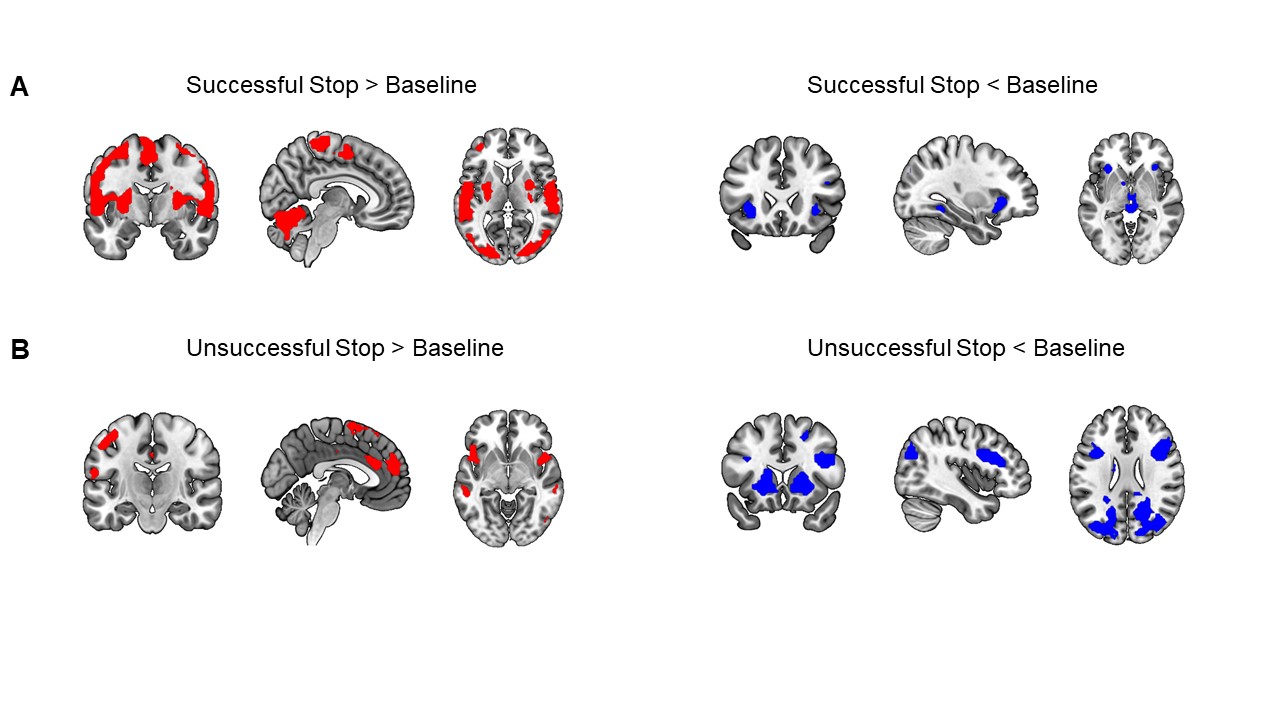
**Figure S3.** Brain activation during successful stop trials versus baseline (A) and unsuccessful stop trials versus baseline (B). All results were corrected for family-wise error (p <0.05).

**Overlap between Adversity and Task Effects**

In terms of adversity and task effect overlap, we found that adversity-related middle temporal gyrus and insula activation were present in both successful and unsuccessful trials compared to baseline (Figure S4). However, the overlap between the adversity and task effect in bilateral MTG was more apparent in the successful stop trials compared to baseline, whereas the overlap between the adversity and task effect in left insula was more apparent in the unsuccessful stop trials compared to baseline. Right insula showed comparable overlap during the both conditions compared to baseline, although it was more activated in the unsuccessful stop trials compared to successful stop trials (Figure S5). Adversity related dACC/superior medial prefrontal cortex activation overlapped only with the task effect during unsuccessful stop trials compared to baseline. No dACC activation was found during successful stop trials versus baseline. Adversity related IFG activation did not overlap with any of the task contrast.


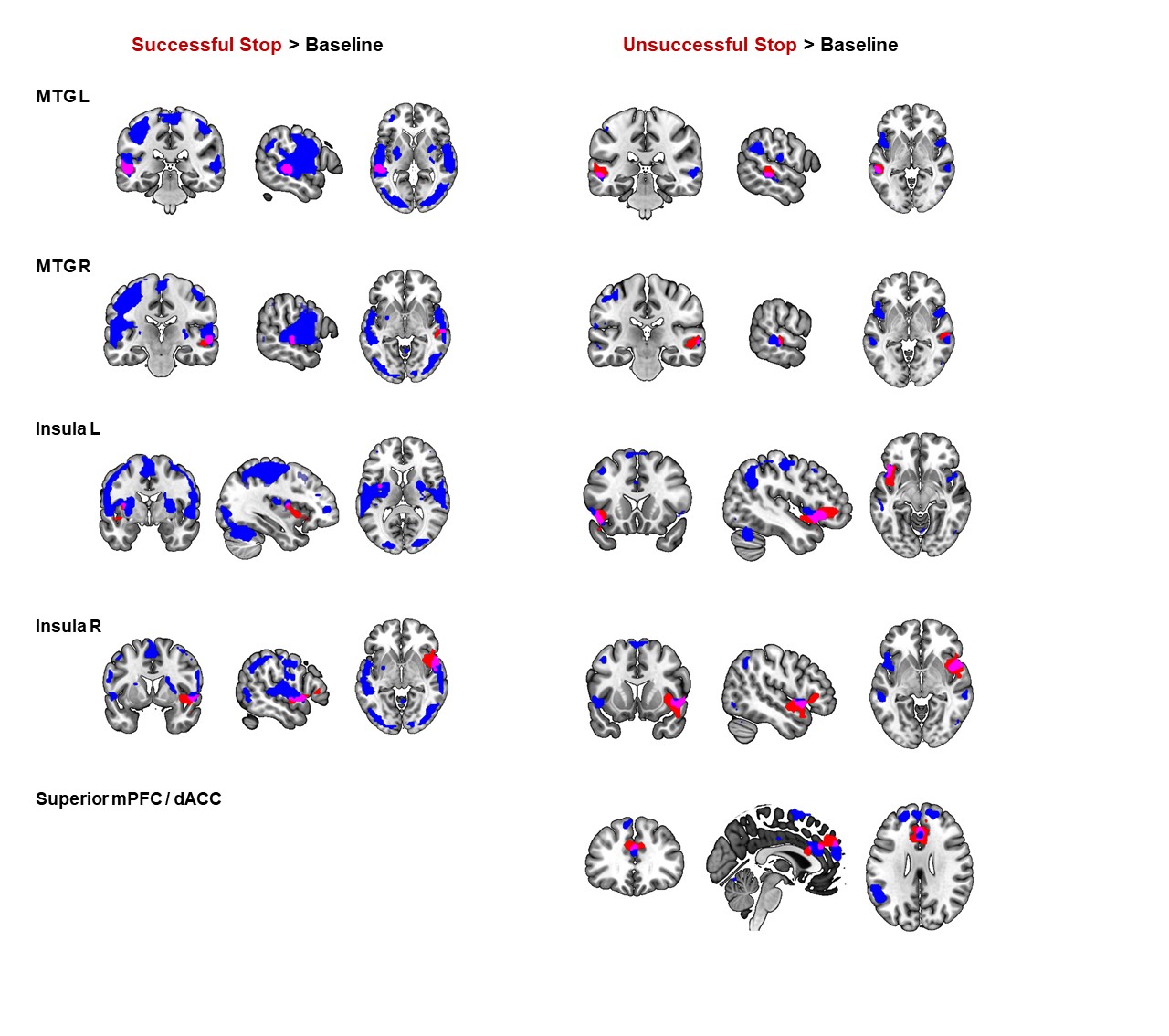
**Figure S4.** Overlap between the adversity effect and task effect during successful stop trials versus baseline (left panel) and unsuccessful stop trails versus baseline (right panel). Blue, red, and pink colors represents task effect, adversity effect, and the overlap between them respectively.

**
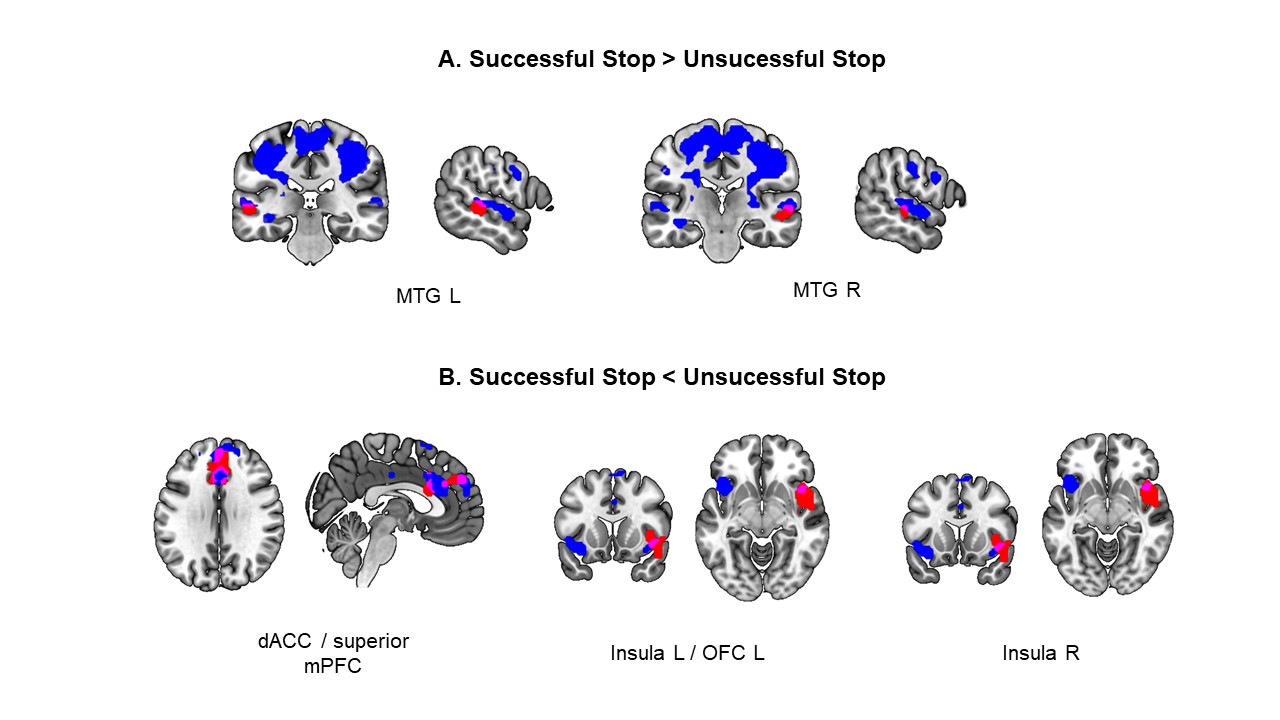
Figure S5.** Overlap between the adversity effect and task effect during successful stop trials versus unsuccessful stop trails. Blue, red, and pink colors represents task effect, adversity effect, and the overlap between them respectively.

# S9. Brain-Behavior Relationship


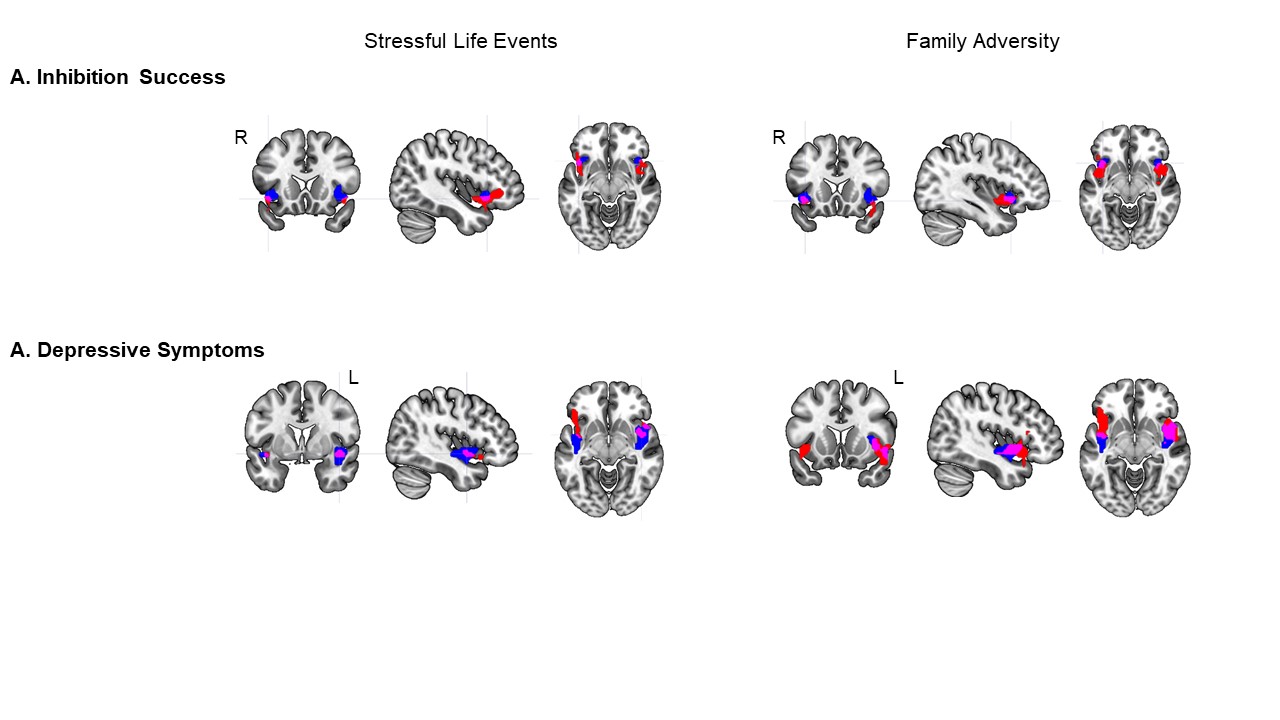


**Figure 6.** The overlap between specific adversities and behavior in bilateral insula. Blue, red, and pink colors represents behavior, adversity effect, and the overlap between them respectively.


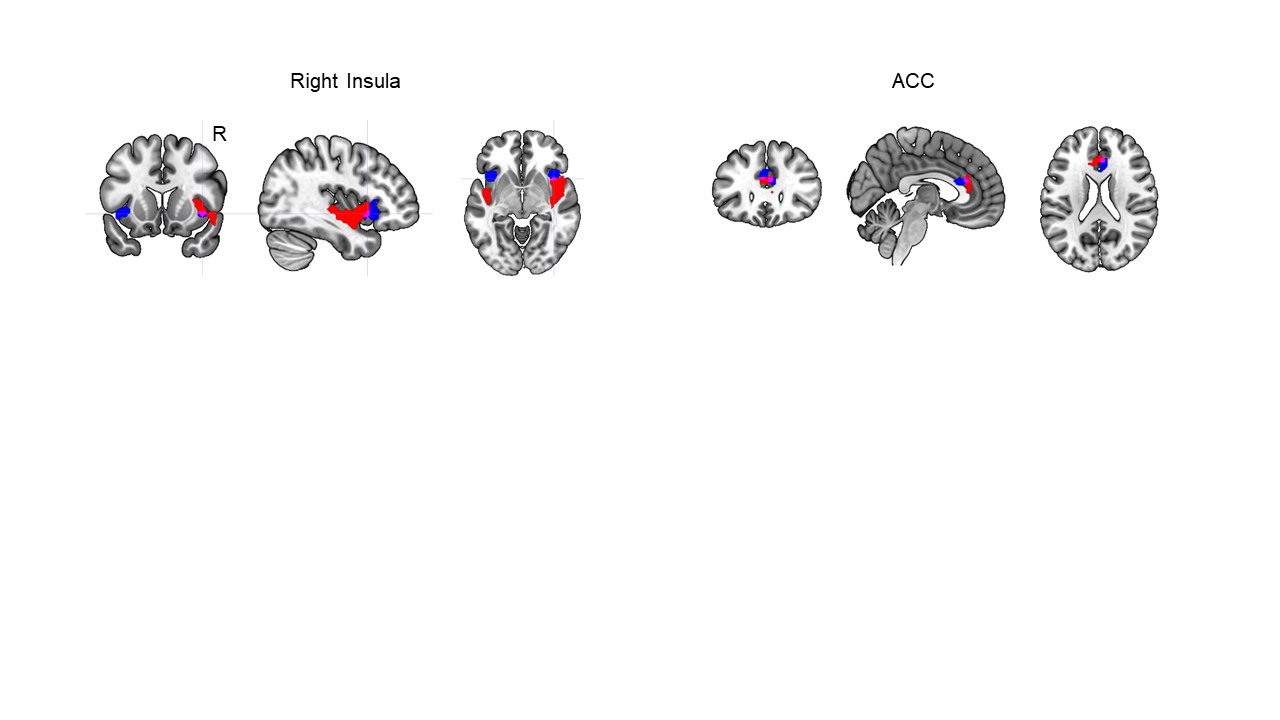


**Figure 7.** The overlap between inhibition success and depressive symptoms during successful versus unsuccessful stop trials. Blue, red, and pink colors represents inhibition success, depressive symptoms, and the overlap between them respectively.

# S10. Neural Correlates of Stop Signal Reaction Time

We conducted a regression analysis in SPM 12 using the stop signal reaction time (SSRT) scores for the successful versus unsuccessful stop trials contrast in which we identified adversity effect. The results are shown in the Figure S8. Higher scores in SSRT was associated with higher activation in several default-mode network regions including left angular gyrus extending middle occipital gyrus (T=5.71, k=296), right angular gyrus (T=5.00, k=105), posterior cingulate cortex /precuneus (T=5.28, k=358) and medial orbitofrontal cortex (T=4.39, k=71) during successful versus unsuccessful stop trials (p < 0.001 for cluster-forming threshold, p <0.05, cluster-level FWE corrected). On the other hand, higher scores in SSRT was associated with lower activation in right insula and inferior frontal gyrus (T=4.89, k=91) during successful versus unsuccessful stop trials (p < 0.001 for cluster-forming threshold, p <0.05, cluster-level FWE corrected).


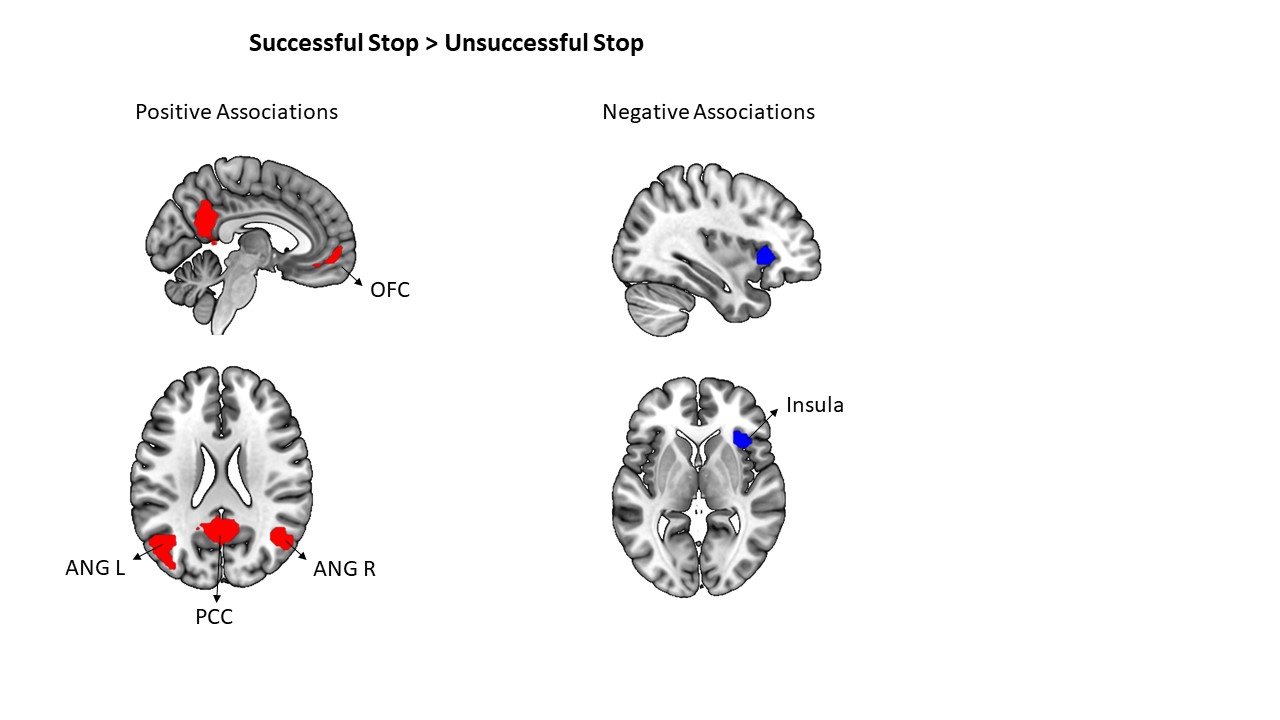


**Figure S8.** Neural correlates of stop signal reaction time during successful versus unsuccessful stop trials. Abbreviations: ANG, angular gyrus; OFC, orbitofrontal cortex; PCC, posterior cingulate cortex.

# S11. Timing Effect of Adversities

Family adversity and stressful life events were assessed at multiple time points across development. We measured family adversity from 3 months (T1) to 11 years (T5) across five assessment waves. It included 11 adverse family factors (e.g., parental psychopathology, marital discord, poor coping skills of parents etc.) at T1. Two items were excluded (unwanted pregnancy and early parenthood) at the following assessment waves since they were specific to T1 measurement. We measured stressful life events, the presence of several life stressors in different domains (partnership, work, health etc.), using the adapted version of the Munich Event List (3) across 11 assessment waves. We calculated Z-transformed scores for each time point and used these time-specific sum scores for the current analysis.

To examine if there is a time window of increased vulnerability to family adversity and stressful life events in the context of inhibitory control, we conducted several regression analyses. All analyses were controlled for sex and current psychopathology. P was set to 0.001 to identify clusters at whole-brain and the identified clusters were reported if p < 0.05 family-wise error corrected at cluster level.

There were significant strong positive correlations between family adversity measures at different time points (Table S6), whereas stressful life events showed small to moderate positive correlations between time points (Table S7).

During successful versus unsuccessful stop trials, family adversity was associated with higher activation in brainstem at T1 (3 months), higher activation in bilateral insula at T2 (2 years) and T3 (4.5 years) and higher activation in left insula at T4 (8 years) and T5 (11 years) (all p < 0.05 FWE-corrected at cluster level; Table S8 and Figure S9). Stressful life events were linked to higher activation in MTG at T2, higher activation in left insula\IFG activation at T5, higher activation in dACC, pregenual ACC, middle cingulum, left caudate, and left angular gyrus at T7 (19 years), and higher activation in dACC and middle cingulum at T9 (23 years) during successful versus unsuccessful stop trials (all p < 0.05 FWE-corrected at cluster level; Table S9 and Figure S9).

**Table S6.** The Spearman’s correlations between family adversity measures.

|  | T1 | T2 | T3 | T4 | T5 |
| --- | --- | --- | --- | --- | --- |
| T1 | - | .69*** | .63*** | .59*** | .52*** |
| T2 |  | - | .71*** | .55*** | .52*** |
| T3 |  |  | - | .65*** | .57*** |
| T4 |  |  |  | - | .75*** |
| T5 |  |  |  |  | - |

*** p < 0.001

**Table S7.** The Pearson’s correlations between stressful life events measures.

|  | T1 | T2 | T3 | T4 | T5 | T6 | T7 | T8 | T9 | T10 | T11 |
| --- | --- | --- | --- | --- | --- | --- | --- | --- | --- | --- | --- |
| T1 | - | .50*** | .41*** | .23* | .32**** | .28*** | .22* | .32*** | .18* | .18* | .06 |
| T2 |  | - | .53*** | .34*** | .22* | .21* | .26** | .26** | .21* | .16 | .25** |
| T3 |  |  | - | .44*** | .18* | .16 | .16 | .26** | .26** | .17 | .19* |
| T4 |  |  |  | - | .38*** | .31** | .13 | .14 | .22* | .10 | .04 |
| T5 |  |  |  |  | - | .41*** | .44*** | .24* | .21* | .04 | .10 |
| T6 |  |  |  |  |  | - | .27** | .03 | .02 | .13 | -.06 |
| T7 |  |  |  |  |  |  | - | .42*** | .48*** | .19* | .25** |
| T8 |  |  |  |  |  |  |  | - | .51*** | .37*** | .40*** |
| T9 |  |  |  |  |  |  |  |  | - | .29** | .34*** |
| T10 |  |  |  |  |  |  |  |  |  | - | .38*** |
| T11 |  |  |  |  |  |  |  |  |  |  | - |

*p <0.05 **p<0.01 ***p<0.001

**Table S8.** Timing effect of family adversity on brain responses during inhibitory control (successful versus unsuccessful stop trials)

| Time | Region | k | t | MNI coordinates [x y z] | | |
| --- | --- | --- | --- | --- | --- | --- |
| T1 | Brainstem | 72 | 4.06 | -9 | -37 | -22 |
| T2 | Insula L | 112 | 5.16 | -42 | 5 | -10 |
|  | Insula R | 77 | 4.44 | 48 | 11 | -10 |
| T3 | Insula L | 112 | 5.29 | -39 | 8 | -7 |
|  | Insula R | 97 | 5.02 | 42 | 11 | -7 |
| T4 | Insula L | 70 | 5.95 | -42 | 5 | -10 |
| T5 | Insula L | 86 | 4.83 | -39 | 8 | -7 |

p < 0.05 (FWE-corrected at cluster level).

**Table S9.** Timing effect of stressful life events on brain responses during inhibitory control (successful versus unsuccessful stop trials)

| Time | Region | k | t | MNI coordinates [x y z] | | |
| --- | --- | --- | --- | --- | --- | --- |
| T2 | MTG L | 89 | 5.19 | -54 | -31 | -1 |
| T5 | Insula L | 75 | 4.18 | -45 | 26 | -4 |
| T7 | pgACC | 96 | 5.01 | -9 | 41 | 14 |
|  | dACC | 360 | 4.96 | -6 | 38 | 38 |
|  | ANG L | 120 | 4.34 | -39 | -73 | 41 |
|  | MCC | 103 | 4.27 | -6 | -34 | 35 |
|  | Caudate L | 121 | 4.20 | -12 | 14 | 2 |
| T9 | dACC | 81 | 4.24 | -9 | 32 | 29 |
|  | MCC | 98 | 3.86 | 9 | -46 | 38 |

p < 0.05 (FWE-corrected at cluster level). Abbreviations: ANG, angular gyrus; dACC, dorsal anterior cingulate cortex; MCC, middle cingulate cortex; pgACC, pregenual anterior cingulate cortex.


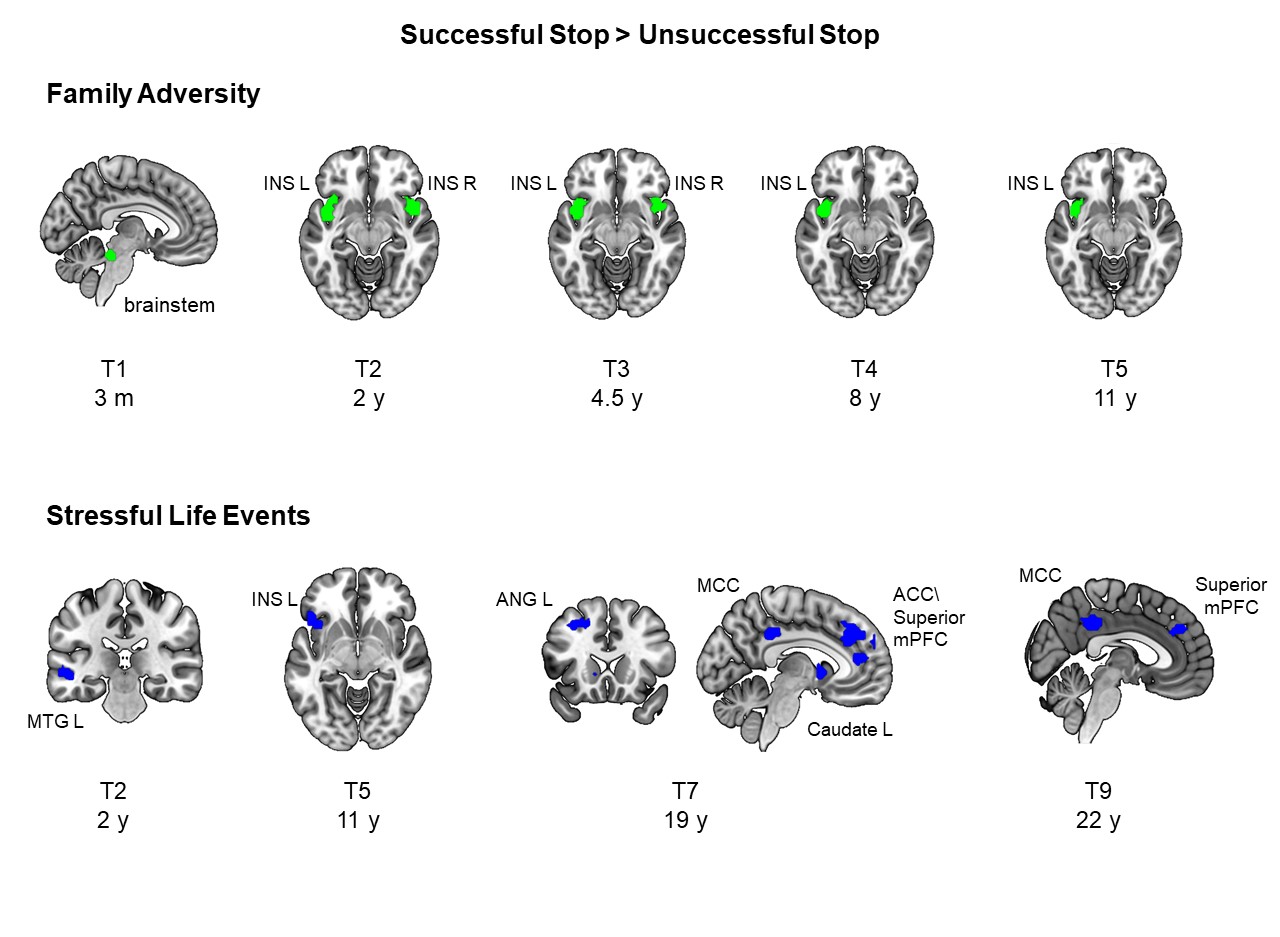


**Figure S9.** Timing effect of prospectively collected adversities on brain responses during successful versus unsuccessful stop trials (p <0.05, FWE corrected at cluster level). Abbreviations: ACC, anterior cingulate cortex; ANG, angular gyrus; INS, insula; MCC, middle cingulate cortex; mPFC, medial prefrontal cortex; MTG, middle temporal gyrus.

References

1. Verbruggen F, Aron AR, Band GPH, Beste C, Bissett PG, Brockett AT, Brown JW, Chamberlain SR, Chambers CD, Colonius H, et al. A consensus guide to capturing the ability to inhibit actions and impulsive behaviors in the stop-signal task. *Elife* (2019) 8:1–26. doi: 10.7554/eLife.46323

2. Rubia K, Smith AB, Brammer MJ, Taylor E. Right inferior prefrontal cortex mediates response inhibition while mesial prefrontal cortex is responsible for error detection. *Neuroimage* (2003) 20:351–358. doi: 10.1016/S1053-8119(03)00275-1

3. Maier-Diewald W, Wittchen H-U, Hecht H, Werner-Eilert K. *Die Münchner Ereignisliste (MEL) - Anwendungsmanual*. München (1983).
